# Comparative evaluation of *Salmonella* Typhimurium vaccines derived from UK-1 and 14028S: Importance of inherent virulence

**DOI:** 10.1371/journal.pone.0203526

**Published:** 2018-09-07

**Authors:** Shilpa Sanapala, Leandra Mosca, Shifeng Wang, Roy Curtiss

**Affiliations:** Department of Infectious Disease and Immunology, College of Veterinary Medicine, University of Florida, Gainesville, Florida, United States of America; Instituto Butantan, BRAZIL

## Abstract

The initial virulence and invasiveness of a bacterial strain may play an important role in leading to a maximally efficacious attenuated live vaccine. Here we show that χ9909, derived from *Salmonella* Typhimurium UK-1 χ3761 (the most virulent *S*. Typhimurium strain known to us), is effective in protecting mice against lethal UK-1 and 14028S (less virulent *S*. Typhimurium strain) challenge. As opposed to this, 14028S-derived vaccine χ12359 induces suboptimal levels of protection, with survival percentages that are significantly lower when challenged with lethal UK-1 challenge doses. T-cell assays have revealed that significantly greater levels of Th1 cytokines IFN-γ and TNF-α were secreted by stimulated T-lymphocytes obtained from UK-1(Δ*aroA*) immunized mice than those from mice immunized with 14028S(Δ*aroA*). In addition, UK-1(Δ*aroA*) showed markedly higher colonizing ability in the spleen, liver, and cecum when compared to 14028S(Δ*aroA*). Enumeration of bacteria in fecal pellets has also revealed that UK-1(Δ*aroA*) can persist in the host for over 10 days whereas 14028S(Δ*aroA*) titers dropped significantly by day 10. Moreover, co-infection of parent strains UK-1 and 14028S resulted in considerably greater recovery of the former in multiple mucosal and gut associated lymphatic tissues. Mice immunized with UK-1(Δ*aroA*) were also able to clear UK-1 infection remarkably more efficiently from the target organs than 14028S(Δ*aroA*). Together, these results provide ample evidence to support the hypothesis that attenuated derivatives of parent strains with higher initial virulence make better vaccines.

## Introduction

*Salmonella enterica* serovar Typhimurium is a Gram-negative, facultative anaerobic pathogen that causes substantial burden of disease globally [1, 2]. Unlike the typhoidal serovars *Salmonella* Typhi and *Salmonella* Paratyphi A that are human-restricted [3], *S*. Typhimurium is a non-typhoidal serovar (NTS) with a diverse host range [3, 4]. Due to its ability to invade and adapt to a wide range of agriculturally important hosts including pigs, poultry, calves, and sheep [4], *S*. Typhimurium-mediated food-borne illnesses are an international public health concern. Although vaccination is one of the best prophylactic measures to control *Salmonella* infection, a licensed vaccine against *S*. Typhimurium for human use does not exist [5]. A multitude of vaccine development endeavors utilizing attenuated live bacteria, whole-cell killed vaccines, and subunit vaccines aimed at inducing long-term, cross protective immunity against multiple *Salmonella* NTS serovars are underway [6–10]. The ability of live attenuated *Salmonella* strains to stimulate strong humoral and cellular immunity has rendered them as an attractive option for development as homologous vaccines and for use as carriers of heterologous antigens [11–14].

Our group has devised several modulating strategies to develop recombinant attenuated *Salmonella* vaccines (RASVs) with enhanced safety, efficacy and biological containment [15–18]. Delayed onset of attenuation strategy ensures that the vaccine strain maintains wild-type phenotype until achieving colonization in effector lymphoid tissues but becomes self-limiting upon delivering protective antigens using a balanced-lethal vector-host system [18–20]. *S*. Typhimurium strain UK-1 (ATCC 68169) is the wild-type parent strain used as the foundation for the majority of the attenuated recombinant and non-recombinant vaccine derivatives studied in our laboratory [21]. UK-1 strain χ3761 was the parent strain from which many commercial vaccines for poultry such as Megan®Egg and Megan®Vac were also derived [21–25]. Since UK-1 is highly pathogenic in several hosts including mice and chickens [21, 26–28], these vaccine derivatives, when orally administered, are presumed to be more immunogenic and hence trigger a greater level of protective immunity than vaccine derivatives from less-virulent *S*. Typhimurium strains 14028S and SL1344 [26, 29]. Previous work in our laboratory involving characterization of UK-1 and SL1344 mutants with deletions of the *crp* and *cdt* genes has demonstrated that UK-1 derived Δ*crp* and Δ*(crp-cdt)* strains conferred complete protection against challenge with its own parent as well as with the less-virulent *S*. Typhimurium strains [30]. We have also utilized comparative genome analysis in a different study [28] to examine the phenotypic impact of genomic differences between UK-1 and other *S*. Typhimurium strains. Significant differences in genomic features which might contribute to virulence were identified between UK-1 and four other strains, LT2, 14028S, D23580, and SL1344.

In the present study, we compared the immunogenicity and protective efficacy of a single-dose oral administration of two *aroA*-deficient strains χ9909 and χ12359, derived from UK-1 χ3761 and 14028S, respectively. UK-1 and 14028S are highly similar, virulent, non-host-adapted strains, although the former has 4-fold lower LD_50_ (2.5 x 10^4^ CFU) than the latter (9.6x10^4^ CFU) [28, 30]. AroA is part of the shikimate pathway, and is involved in the synthesis of aromatic amino acids and several vitamins. As these aromatic amino acids and vitamins are not readily available in the mammalian host, lack of *aroA* results in auxotrophy and attenuation of *Salmonella* [31–33]. Hence, such attenuated metabolic mutants of *Salmonella* can be used as potential vaccine candidates against lethal homologous and heterologous infections [34, 35]. In this capacity, the current study investigates the magnitude of protection offered by the two strains UK-1(Δ*aroA*) and 14028S(Δ*aroA*) against lethal challenge with both parent strains, in a dose-dependent fashion. In addition to survival rate, these *aroA*-deficient strains were also evaluated in terms of antibody responses, cell-mediated cytokine production, pathogen clearance, and colonization of intestinal mucosa and deeper tissues. Findings reported in this paper provide evidence to further corroborate the notion that attenuated vaccines derived from hypervirulent strain UK-1 induce protective immunity that is markedly superior to those derived from the less-virulent *S*. Typhimurium strains such as 14028S.

## Results

### Evaluation of protective immunity

**Fig 1:**
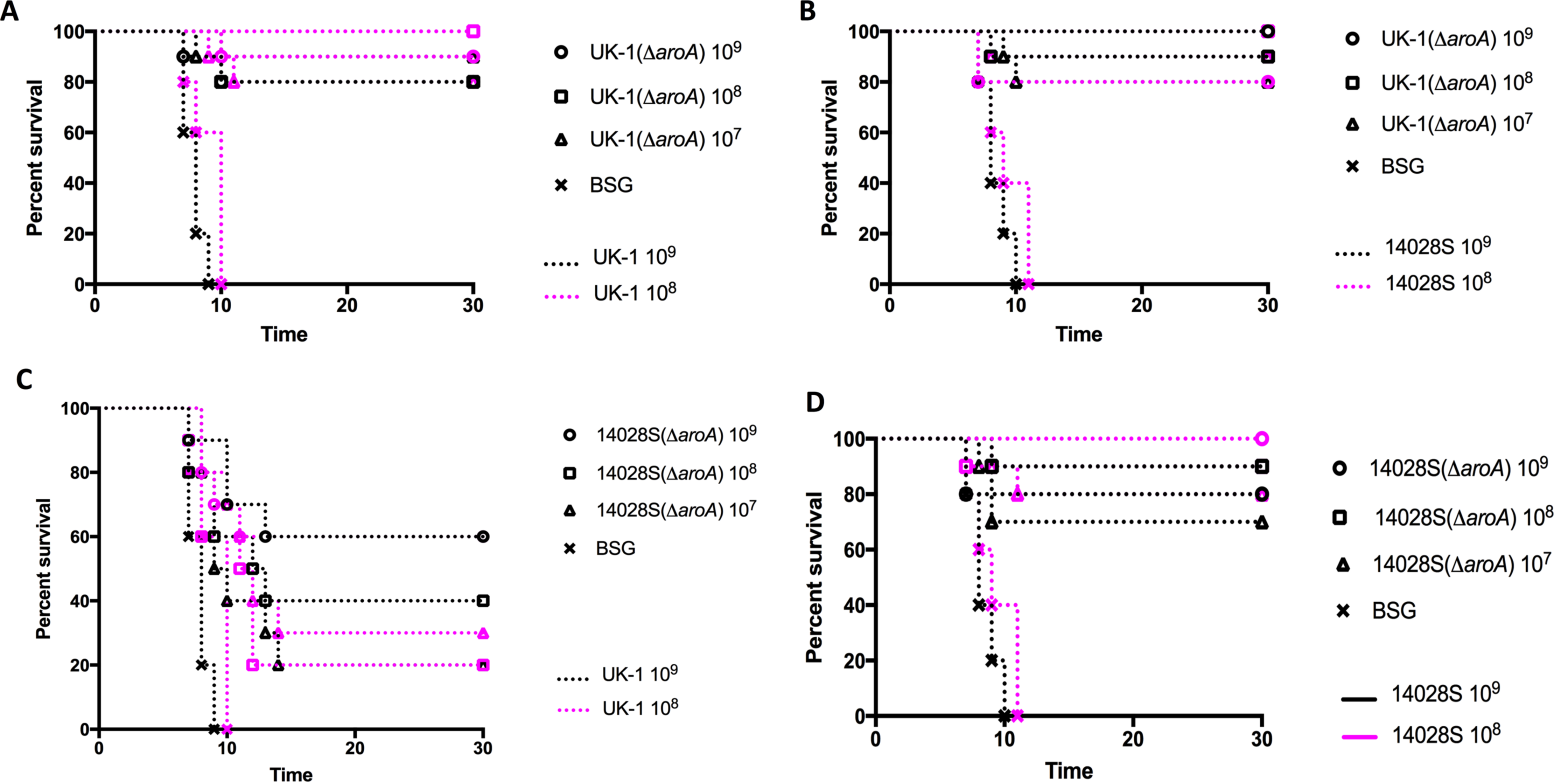
Protective efficacy of Δ*aroA* mutants UK-1(Δ*aroA*) and 14028S(Δ*aroA*) against UK-1 and 14028S challenge. BALB/c mice (*n* = 10 per immunization dose) were orally immunized with UK-1(Δ*aroA*) or 14028S(Δ*aroA*) at doses 1x10^9^, 1x10^8^ or 1x10^7^ CFU in 20 μl BSG. Mice were also mock-vaccinated with 20 μl BSG (n = 5). Oral challenge at doses 1x10^9^ or 1x10^8^ CFU were performed with UK-1 or 14028S 35 days after immunization. Mice were monitored for mortality and signs of morbidity for 30 days after challenge. Survival curve of (A) UK-1(Δ*aroA*)-vaccinated UK-1 challenged mice, (B) UK-1(Δ*aroA*)-vaccinated 14028S challenged mice, (C) 14028S(Δ*aroA*)-vaccinated UK-1 challenged mice, and (D) 14028S(Δ*aroA*)-vaccinated 14028S challenged mice. Log-rank (Mantel-Cox) test was used to plot and analyze survival curves.

Given that UK-1 χ3761 was more highly virulent when compared to 14028S [28], we sought to determine whether immunization with its attenuated derivative UK-1(Δ*aroA*) induces a greater level of protective immunity against lethal oral challenge than strain 14028S(Δ*aroA*). To this end, BALB/c mice were orally immunized with 1x10^9^, 1x10^8^ or 1x10^7^ CFU doses of UK-1(Δ*aroA*) or 14028S(Δ*aroA*). 35 days after immunization, mice were orally challenged with a 1x10^9^ (~10^5^ x LD_50_) or 1x10^8^ (~10^4^ x LD_50_) CFU dose of parent strains UK-1 or 14028S. A schematic of the vaccination schedule is presented in S1 Fig. We observed consistently higher survival percentages (80–100%) against both challenge strains and challenge doses in mice that were immunized with UK-1(Δ*aroA*) (Fig 1A and 1B). In contrast, varying levels of protection ranging from 20–100% were observed in mice immunized with 14028S(Δ*aroA*) (Fig 1C and 1D). The survival rate appeared to be dose dependent. Specifically, the 1x10^7^ CFU immunizing dose of 14028S(Δ*aroA*) conferred only 20 and 30% protection against 1x10^9^ (*P* < 0.01) and 1x10^8^ (*P* < 0.05) CFU challenge doses of UK-1, respectively (Fig 1C). The 1x10^8^ CFU dose of 14028S(Δ*aroA*) also yielded suboptimal levels of protection ranging between 20–40% against UK-1 challenge (*P* < 0.001). 60% survival was observed with 1x10^9^ CFU of 14028S(Δ*aroA*) against both 1x10^9^ and 1x10^8^ CFU doses of UK-1, which is still lower, although not significantly so, than the 80–100% protection offered against 14028S challenge (Fig 1D). Evidently, 14028S(Δ*aroA*) provided reasonably good percentages of protection (70–100%) at all doses against 14028S challenge (Fig 1D) that were better than against UK-1 challenge. These results suggest that UK-1(Δ*aroA*) could effectively protect immunized mice at all doses against both UK-1 and 14028S challenge. Contrary to this, although 14028S(Δ*aroA*) performed well against challenge with its parent 14028S, it failed to elicit optimal protection against challenge with the more virulent UK-1, specifically at lower immunization doses.

### Antibody responses to SOMPs and LPS

**Fig 2:**
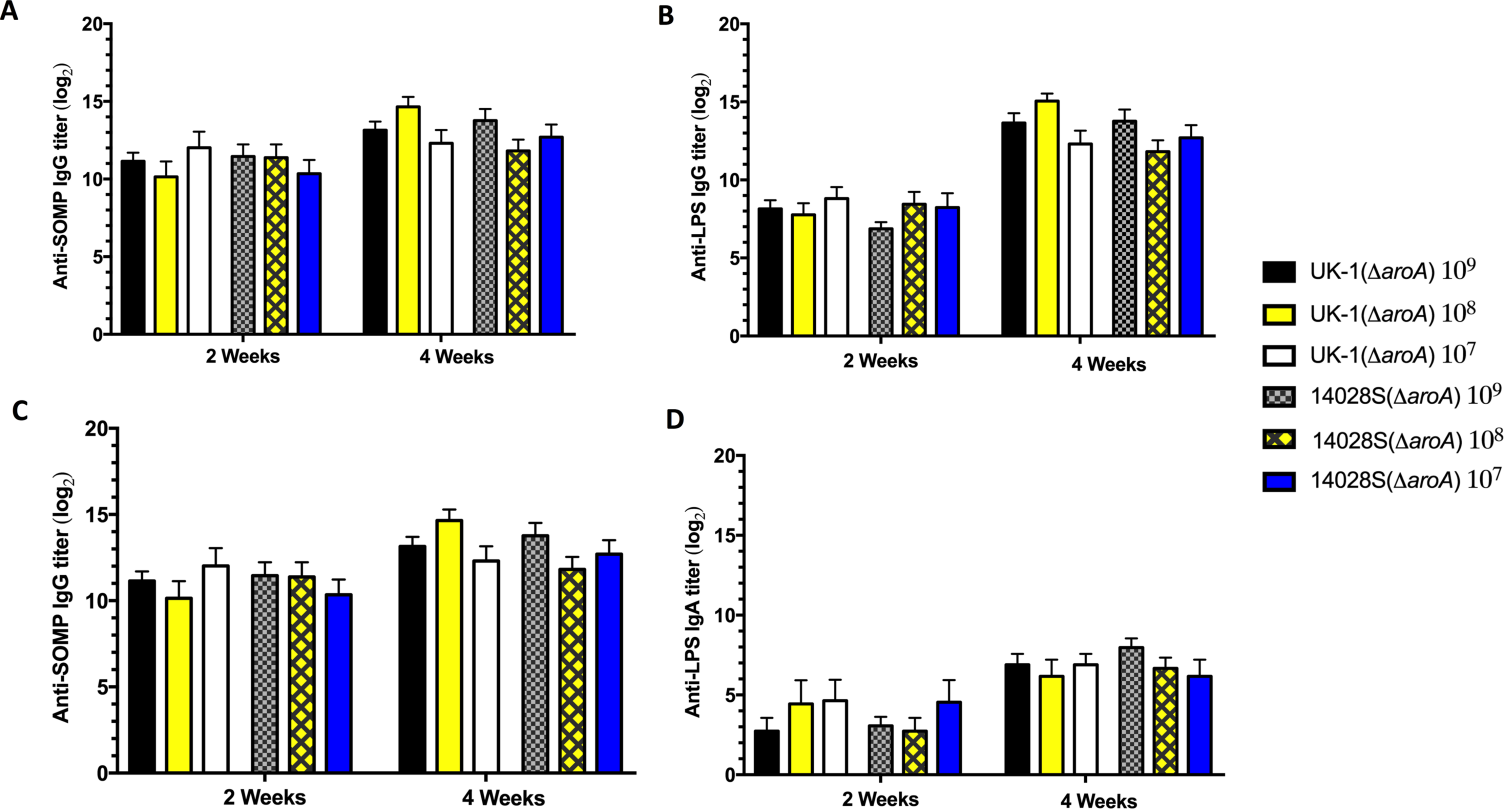
Anti-SOMP and anti-LPS antibody responses in mice. Serum IgG responses to (A) SOMP, and (B) LPS as well mucosal IgA responses to (C) SOMP, and (D) LPS were determined by ELISA at 2 and 4 weeks after oral immunization in sera and vaginal washes from BALB/c mice (*n* = 6) orally immunized with UK-1(Δ*aroA*) or 14028S(Δ*aroA*) at doses 1x10^9^, 1x10^8^ or 1x10^7^ CFU. No statistically significant difference was observed between vaccine groups.

Serum immunoglobulin G (IgG) and mucosal IgA responses to *Salmonella* outer membrane proteins (SOMPs) and lipopolysaccharide (LPS) were measured by ELISA (Fig 2A–2D). Both *aroA* mutants UK-1(Δ*aroA*) and 14028S(Δ*aroA*) induced equivalent anti-SOMP and anti-LPS serum IgG responses (Fig 2A & 2B). At 4 weeks, the IgG titers in mice immunized with either UK-1(Δ*aroA*) or 14028S(Δ*aroA*) were slightly higher than those at 2 weeks, with no significant differences between the groups. Additionally, the secretory IgA endpoint titers induced by both vaccine strains were suboptimal at both 2 and 4 weeks (Fig 2C & 2D). No serum IgG or secretory IgA titers were detected in mice that were mock-vaccinated with BSG, as expected (data not shown). The type of immune response to SOMPs and LPS was determined by measuring the levels of IgG isotype subclasses IgG1 and IgG2a (S2A–S2D Fig). A Th1- and Th2-type mixed response was observed against LPS antigen since both strains induced high IgG1 and IgG2a responses. The IgG2a titers to SOMP at 4 weeks in both UK-1(Δ*aroA*) (*P* < 0.001) and 14028S(Δ*aroA*) (*P* < 0.0001) groups were significantly higher than IgG1 titers. Collectively, these data suggest that there is no difference in antibody responses between UK-1(Δ*aroA*) and 14028S(Δ*aroA*)-immunized mice, suggesting that antibodies might play a role in vaccine-mediated protection against challenge with wild-type parent strains, but are not the sole determining factor.

### Antigen presentation and cytokine production

**Fig 3:**
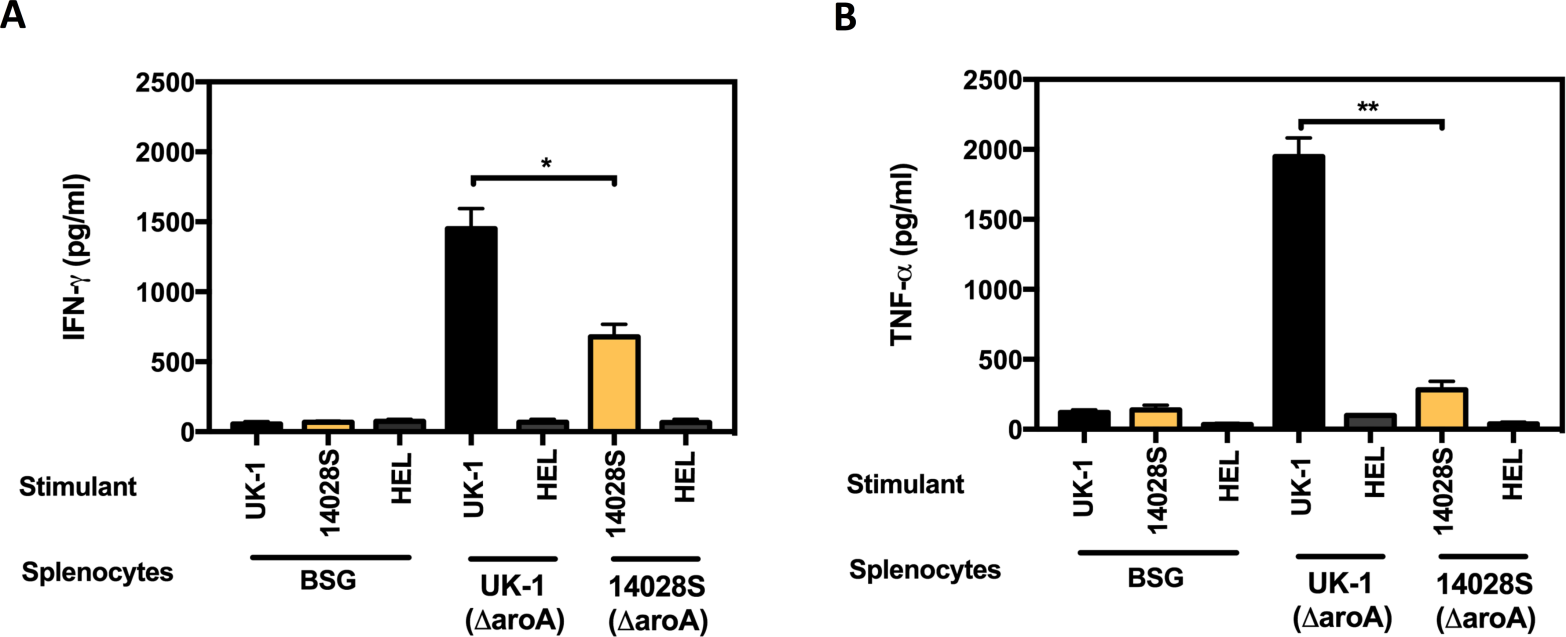
APC-mediated T-cell activation and cytokine secretion. T-lymphocytes obtained from BALB/c mice (*n* = 5) orally immunized with UK-1(Δ*aroA*), 14028S(Δ*aroA*) or BSG (mock) were co-incubated with respective UV-inactivated UK-1 or 14028S-infected and mitomycin C—treated APCs. APCs treated with an unrelated antigen, HEL, were also included for comparison. The co-cultures were incubated for 72 h and supernatants were collected for determination of (A) IFN-γ, (B) TNF-α. *P ≤ 0.05, **P ≤ 0.01.

Since both UK-1(Δ*aroA*) and 14028S(Δ*aroA*) elicited similar levels of antibody responses despite differential protective efficacies, cytokine concentrations were assayed to compare with protection data. Antigen-presenting cells (APCs) were generated by treating the spleen cells obtained from naïve BALB/c mice with mitomycin C, followed by removal of non-adherent cells. APCs (2 x 10^5^ cells/well) were then infected with UV-inactivated UK-1 or 14028S (MOI of 10) for 2 h. Hen egg lysozyme (HEL) was included as an unrelated antigen for comparison. These antigen-loaded APCs were co-cultured for 72 h with T-lymphocytes obtained from spleens of orally immunized mice (UK-1(Δ*aroA*) or 14028S(Δ*aroA*)). T-cells obtained from the BSG (mock-vaccinated) group were also co-cultured with *Salmonella* or HEL treated APCs as control. IFN-γ, TNF-α, IL-4 and IL-12 levels in the culture supernatants were assayed following incubation. As shown in Fig 3A and 3B, T-lymphocytes obtained from BSG group when co-cultured with APCs previously infected with *Salmonella* or HEL, had minimal cytokine induction. In contrast, T-lymphocytes from mice primed by oral vaccination with UK-1(Δ*aroA*) exhibited markedly elevated levels of IFN-γ and TNF-α upon incubation with APCs infected with UV-inactivated UK-1. However, T-lymphocytes from mice similarly immunized with 14028S(Δ*aroA*) and co-cultured with APCs previously infected with UV-inactivated 14028S showed significantly lower levels of IFN-γ (*P* < 0.05) and TNF-α (*P* < 0.01) in the supernatants compared with UK-1(Δ*aroA*). In addition, incubation of UK-1 or 14028S infected APCs with T-cells from mice immunized with respective mutant strains also did not cause significant induction of IL-4 or IL-12 (S3A and S3B Fig). Thus, proinflammatory responses such as increased secretion of IFN-γ and TNF-α appear to contribute positively to the observed superior protective efficacy of UK-1(Δ*aroA*).

### Co-infection with UK-1 and 14028S

**Fig 4:**
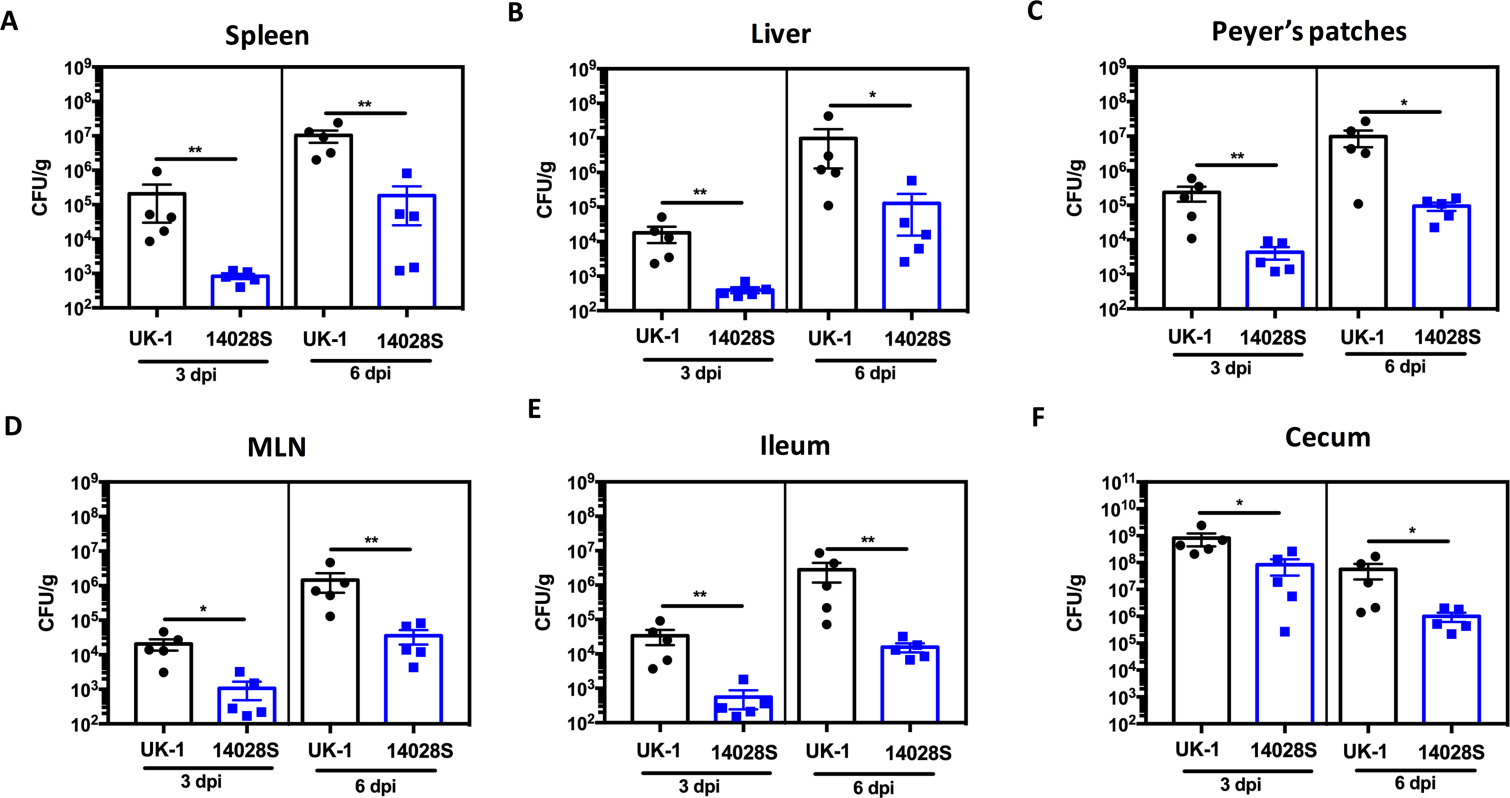
Co-infection with UK-1 and 14028S. BALB/c mice were orally challenged with a 1:1 mixture of 1 x 10^9^ CFU of UK-1(Nal^r^) and 14028S(Cm^r^ Tc^r^). Groups of mice (n = 5 per group) were euthanized on days 3 and 6 post challenge. (A) Spleen, (B) liver, (C) Peyer’s patches, (D) MLN, (E) ileum, and (F) cecum were collected to determine the bacterial burdens. Significantly higher UK-1 burdens were observed in all tested organs on both day 3 and 6 compared to 14028S burdens. *P ≤ 0.05, **P ≤ 0.01.

**Fig 5:**
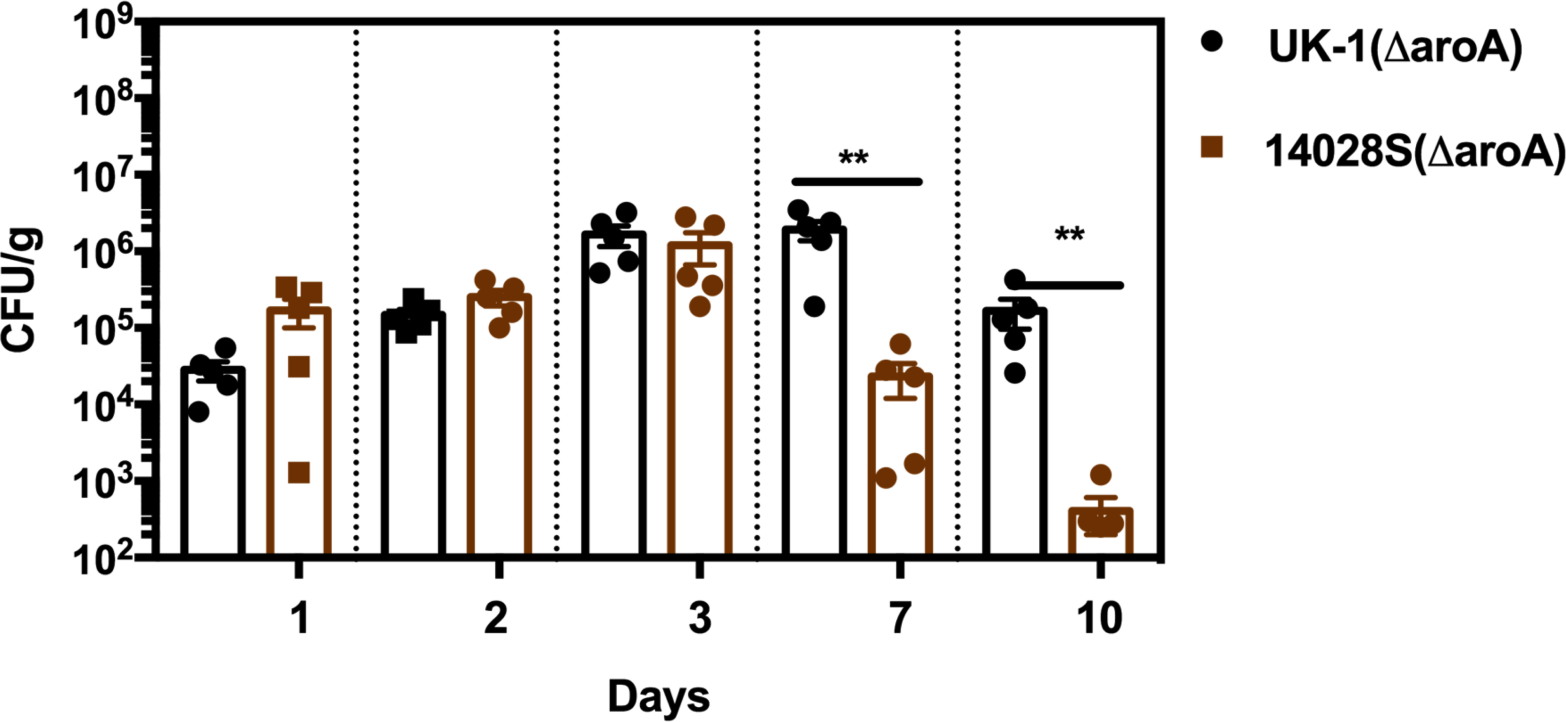
Vaccine strain detection in fecal pellets following oral immunization. BALB/c mice (*n* = 5) were orally immunized with UK-1(Δ*aroA*), 14028S(Δ*aroA*) or BSG (mock). Fecal pellets were collected on days 0, 1, 2, 3, 7, and 10 post immunization for enumeration of bacterial shedding post vaccination. 14028S(Δ*aroA*) titers in the fecal pellets declined significantly by day 7 and continued to decline at day 10. Mice vaccinated with UK-1(Δ*aroA*) maintained significantly higher bacterial titers (**P ≤ 0.01) by day 10 post immunization (last time-point analyzed) when compared to 14028S(Δ*aroA*)-vaccinated mice.

UK-1(Nal^r^) and 14028S(Cm^r^ Tc^r^) were mixed equivalently and inoculated orally into mice (1 x 10^9^ CFU/mouse) to test the fitness of the two strains directly against each other. Bacteria were enumerated in spleen, liver, Peyer’s patches, MLN, cecum, and ileum on days 3 and 6 post infection. Strains were distinguished phenotypically by using antibiotic markers. UK-1(Nal^r^) outcompeted 14028S(Cm^r^ Tc^r^) and hence, a significantly higher UK-1 burden was observed (*P* < 0.05) in gastrointestinal tissues as well as systemic sites on both 3 and 6-day time points post infection when compared to 14028S (Fig 4A–4F). Nevertheless, when colonization was compared by infecting the mice with single strains, 14028S colonized as efficiently as UK-1 in all the tissues (S4A–S4F Fig). Conversely, when colonization was compared by inoculating the mice with UK-1(Δ*aroA*) or 14028S(Δ*aroA*), UK-1(Δ*aroA*) showed higher burdens in systemic tissues compared to 14028S(Δ*aroA*) (spleen *P* < 0.01, liver *P* < 0.01). Higher UK-1(Δ*aroA*) titers were also found in cecum at 6 days post infection (*P* < 0.01) (S5A–S5F Fig). Interestingly, 14028S(Δ*aroA*) was shed at significantly reduced numbers in feces by day 7 (*P* < 0.01) when compared to UK-1(Δ*aroA*) (Fig 5).

### UK-1 burden following immunization with UK-1(Δ*aroA*) and 14028S(Δ*aroA*)

**Fig 6:**
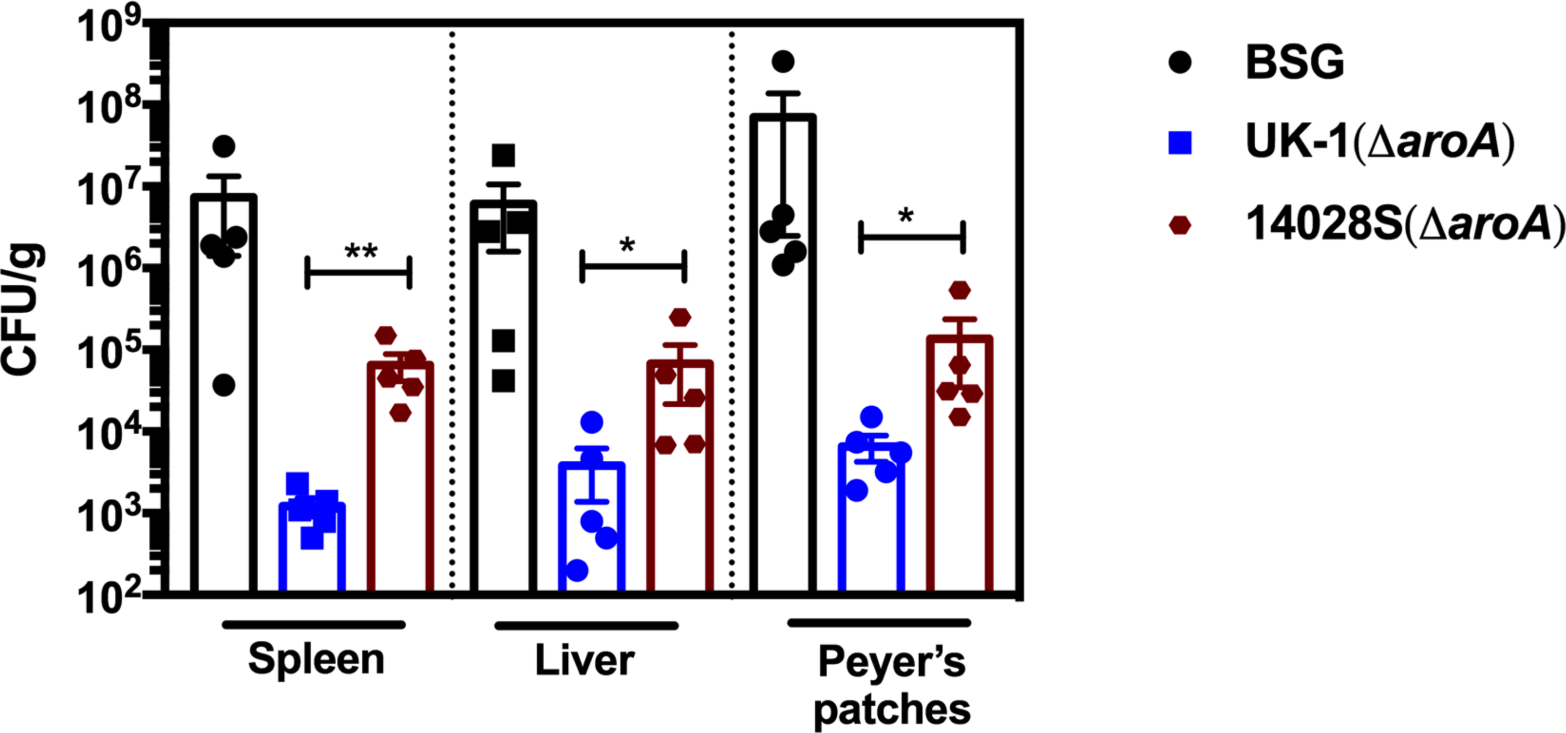
UK-1 burdens are controlled efficiently by UK-1(Δ*aroA*), but not 14028S(Δ*aroA*). BALB/c mice (*n* = 5) were orally immunized with UK-1(Δ*aroA*), 14028S(Δ*aroA*) or BSG (mock) on day 0 and orally challenged with UK-1 or 14028S on day 35. Spleen, liver, and Peyer’s patches were collected on day 6 post challenge to determine the bacterial burdens. UK-1 burdens were significantly reduced in all target organs when mice were immunized with UK-1(Δ*aroA*), but not when immunized with 14028S(Δ*aroA*), as compared to mock-vaccinated mice. *P ≤ 0.05, **P ≤ 0.01.

Since UK-1(Δ*aroA*) and 14028S(Δ*aroA*) showed differential levels of protection to UK-1 challenge, the ability of these strains in clearing UK-1 from various organs was tested. When mice were immunized with a 1x10^9^ CFU dose of UK-1(Δ*aroA*) or 14028S(Δ*aroA*) and challenged 35 days after immunization, significantly higher bacterial burdens were observed after 6 days in all 14028S(Δ*aroA*) immunized mouse tissues that were enumerated (spleen (*P* < 0.01), liver (*P* < 0.05), and Peyer’s patches (*P* < 0.05)) compared to in UK-1(Δ*aroA*) immunized mouse tissues (Fig 6). Consistently high UK-1 burdens were also observed in all organs of the BSG control group mice. Thus, UK-1(Δ*aroA*)-immunized mice showed significant restriction of UK-1 spread in all target organs compared to 14028S(Δ*aroA*) as well as BSG-vaccinated groups.

## Discussion

While much research effort has focused on utilizing various *S*. Typhimurium strains to develop vaccines against salmonellosis and a variety of other infectious diseases, the initial virulence of the parent strain is seldom considered important in predetermining a vaccine’s efficacy. Two previous studies from our laboratory [28, 30] have suggested that the hypervirulent nature of UK-1 might impart its attenuated derivatives an advantage over similarly attenuated mutants derived from relatively less-virulent *S*. Typhimurium strains. The current study is the first report that provides conclusive evidence towards this proposition. We have demonstrated here that a single oral immunization with *aroA*-deficient UK-1 mutant, UK-1(Δ*aroA*), presents a greater level of protection against infection with UK-1 as well as 14028S than afforded by immunization with the less-virulent 14028S(Δ*aroA*) strain. Consistent with this, UK-1(Δ*aroA*)-vaccinated mice displayed significantly reduced UK-1 burden in Peyer’s patches, spleen and liver 6 days after lethal challenge, indicating efficient pathogen clearance. In contrast, the 14028S derivative, 14028S(Δ*aroA*), was effective in protecting against a challenge with its parent, and somewhat effective against a UK-1 challenge. The difference became more prominent at lower doses of immunization. At 10^7^ CFU (low dose) of 14028S(Δ*aroA*), a majority of the mice succumbed to UK-1 infection by day 10, which is likely due to the relatively high UK-1 burden in the target organs.

A similar study conducted by Zhang et al. [30] describes deletions of the *crp* (cyclic AMP receptor protein) and *cdt* (colonization of deep tissues) genes in UK-1 and SL1344. These Δ*crp* and Δ(*crp*-*cdt*) mutants conferred complete protection against challenge with SL1344. However, neither UK-1 nor SL1344 based attenuated strains could fully protect mice against a UK-1 challenge even though the UK-1 attenuated strains performed significantly better than the latter. Furthermore, these Δ*crp* and Δ(*crp*-*cdt*) mutants were also unable to efficiently colonize internal lymphoid organs, suggesting poor induction of systemic or cellular immune responses. On the other hand, the current study illustrates that the UK-1 derived Δ*aroA* mutant UK-1(Δ*aroA*) not only provides complete protection against challenge with the less-virulent 14028S, but also fully protects against UK-1 challenge with a single immunization dose. Moreover, UK-1(Δ*aroA*) efficiently colonizes both gut-associated lymphoid tissue (GALT) as well as deeper tissues such as spleen and liver. While both UK-1(Δ*aroA*) and 14028S(Δ*aroA*) induce strong IgG responses, T-cell assays have shown that only the former could induce high-level secretion of IFN-γ and TNF-α. This trend parallels the poor colonizing ability of 14028S(Δ*aroA*) in internal lymphoid organs as opposed to that of UK-1(Δ*aroA*). Accordingly, colonization of deeper tissues and secretion of proinflammatory cytokines appear to be the underlying mechanisms mediating the induction of seemingly superior protection in UK-1(Δ*aroA*)-immunized mice. Even though both UK-1(Δ*aroA*) and 14028S(Δ*aroA*) could effectively colonize GALT, neither were able to elicit considerable secretory IgA responses. Overall, UK-1(Δ*aroA*) could afford excellent protection despite low mucosal antibody responses, and 14028S(Δ*aroA*) only suboptimal levels of protection even with high levels of IgG, thus antibodies seem not to be the principal basis for protective immunity to *Salmonella* infection.

Cytokine induction in response to *Salmonella* is crucial for the control and resolution of an infection [36–38]. Previous studies have reported that IFN-γ, TNF-α, IL-12, IL-15, IL-18, and IL-1β play a vital role in anti-*Salmonella* defense whereas IL-4 and IL-10 inhibit these protective host defenses [37, 39]. We examined the secretion of IFN-γ, TNF-α, IL-12, and IL-4 in vitro 35 days after immunization. T-lymphocytes obtained from UK-1(Δ*aroA*) immunized mice, upon incubation with APCs that were infected with UV-inactivated UK-1 released significant amounts of IFN-γ and TNF-α into the supernatants. Since IL-12 is known to be essential for promoting type 1 cytokine responses and cell-mediated immunity against *Salmonella* [37], we expected to find significant levels of this cytokine in the supernatants following 72 h incubation. However, we detected only suboptimal amounts of IL-12 upon stimulation with UV-inactivated UK-1 that may be due to the time of assessment of the supernatants. As IL-12 is produced early in the immune response to *Salmonella* infection, maximal levels might have been detected if analyzed at earlier time points. Degree and duration of IL-12 response may have significant consequences in terms of induction of cellular immunity and protection via production of Th1 cytokines. Interestingly, consistent with the mixed Th1/Th2 response exhibited by serum antibodies against LPS and SOMPs, a noticeable amount of IL-4 was also detected in both UK-1(Δ*aroA*) and 14028S(Δ*aroA*) groups upon stimulation. Nevertheless, it is also possible that the cytokine profile could have been considerably different if the mice were euthanized at an earlier time following immunization. However, since the mice were challenged with UK-1 or 14028S 35 days after immunization during survival and burden studies, we chose the day 35 time point for assessing cytokine production as well.

Besides differential Th1 cytokine production and colonization of internal lymphoid organs, another striking difference observed between UK-1(Δ*aroA*) and 14028S(Δ*aroA*) was the cecal and fecal titers following immunization. UK-1(Δ*aroA*) was detected in the fecal pellets for a significantly longer duration than 14028S(Δ*aroA*). This greater level of fecal shedding also correlated with the relatively higher level of cecal colonization as compared to 14028S(Δ*aroA*). However, these observations do not seem to accurately reflect intestinal colonization; as 14028S(Δ*aroA*) also colonizes GALT efficiently although it is unable to disseminate to systemic sites. UK-1(Δ*aroA*) has a significantly greater presence in the ceca and fecal pellets as well as in the deeper tissues, which likely contributes to its protective efficacy. We rule out the possibility that this greater persistence of UK-1(Δ*aroA*) could prevent subsequent colonization of the wildtype challenge strains since UK-1(Δ*aroA*) was not detected in the fecal pellets collected at 2 and 4 weeks post immunization. The hypervirulent nature of UK-1 likely drives these protective phenomena. In this regard, co-infection of UK-1 with 14028S clearly portrayed the ability of UK-1 to outcompete 14028S and aggressively colonize both gastrointestinal and systemic tissues.

A recent study [13] testing the efficacy of UK-1 and 14028S based LPS core deletion (Δ*rfaG*) mutant strains in cancer therapy has demonstrated that the UK-1 background carried a significantly higher therapeutic capacity than the 14028S background. Unlike UK-1(Δ*aroA*) and 14028S(Δ*aroA*) from our study, Δ*rfaG* mutants from both UK-1 and 14028S backgrounds showed similar organ colonization and TNF-α induction. Another study [40] showed that Δ*aroA* strains exhibited increased in vivo immunogenicity and pathogenicity compared to its highly immunogenic parent. However, we did not observe increased virulence or tissue burdens during our studies with Δ*aroA* mutants. One explanation could be the intravenous route of inoculation used in the abovementioned studies. To resolve this discrepancy, further testing is required to investigate whether parenteral administration of Δ*aroA* mutants may lead to increased immunostimulatory capacity. Future studies should also focus on comparing UK-1 and other *S*. Typhimurium based Δ*aroA* vaccine strains containing various other mutations that enable delivery of homologous and heterologous antigens. Additional attenuating mutations will also be introduced to shut off the virulence phenotypes in vivo, in order to enhance the safety of these strains prior to their use as antigen-delivering vectors.

To conclude, results of our study strongly indicate that vaccines derived from highly pathogenic UK-1 achieve maximal immunogenicity and protection when compared to less-virulent *S*. Typhimurium strains. These findings can be applied towards rational vaccine design to select the best vaccine candidates, especially among isogenic strains exhibiting distinct pathogenic properties, for further optimization and clinical testing.

## Materials and methods

### Ethics statement

All procedures involving the use of mice were thoroughly reviewed and approved by the University of Florida, Gainesville IACUC, the Institutional Animal Care and Use Committee (Study #201509049). All protocols conform to the federal regulations and policies outlined by the United States Department of Agriculture, Animal and Plant Health Inspection Service (USDA-APHIS) and Office of Laboratory Animal Welfare (OLAW).

For assessment of survival, death was used as the endpoint for evaluating protective efficacy of the vaccine strains. Specific criteria for establishing early endpoints were discussed and approved by IACUC and animal care representatives to achieve optimal scientific results without compromising conscientious ethical standards. To this end, mice exhibiting signs of distress such as labored breathing, acute dehydration, severe hunching and impaired motility were considered moribund and humanely euthanized within 4 h after onset of symptoms via carbon dioxide asphyxiation followed by cervical dislocation. Professional advice from veterinary technicians was also sought by research staff for mice in question.

### Bacterial strains, plasmids, growth conditions, and reagents

Bacterial strains and plasmids used in this study are listed in Table 1. *aroA* mutants were generated from *S*. Typhimurium using suicide vector pYA3600 [41]. For routine use, *S*. Typhimurium vaccine strains were grown with aeration in LB medium supplemented with 0.1% glucose at 37°C [42]. Strains with the Δ*aroA21419* deletion were grown with the addition of PABA (Para-aminobenzoic acid) and DHB (2,3-dihydroxybenzoic acid) at concentrations of 2 μg/ml. MacConkey plates with 1% lactose, necessary supplements, and antibiotics were used for colonization and coinfection experiments. All media were purchased from BD Difco (Franklin Lakes, NJ) unless otherwise indicated. Antibiotics were added as needed at the following concentrations: chloramphenicol, 20 μg/ml; tetracycline, 50 μg/ml; nalidixic acid, 30 μg/ml. All antibiotics and chemicals were purchased from Sigma (St. Louis, MO) or Fisher Scientific (Pittsburgh, PA).

**Table 1:** Characteristics of the strains and plasmids used in this study.

Strain or plasmid	Relevant Characteristics	Designation	Source
χ3761	UK-1 wild-type *Salmonella* Typhimurium	UK-1	[21]
ATCC 14028	14028S wild-type *Salmonella* Typhimurium	14028S	[43]
χ9909	Δ*aroA21419*, derived from χ3761	UK-1(Δ*aroA*)	[41]
χ12359	Δ*aroA21419*, derived from 14028S	14028S(Δ*aroA*)	Curtiss lab chi collection
χ4138	*gyrA1816*, derived from χ3761	UK-1(Nal^r^)	[43]
pYA3600	Suicide vector for generation of Δ*aroA21419*	-	[41]
pACYC184	*E*. *coli* plasmid cloning vector (p15A *ori*) with chloramphenicol and tetracycline resistance genes	Cm^r^ Tc^r^	[44]

### Mice

Female BALB/c mice were obtained from Charles River Laboratories (Wilmington, MA) and acclimated for 7 days after arrival in the general housing facility. Mice were seven-weeks-old at the time of commencement of the experiments.

### Immunization and challenge

Mice (5 to 10 per group) were deprived of food and water for 6 h before and 30 min after oral immunization. *Salmonella* strains UK-1(Δ*aroA*) and χ14028S(Δ*aroA*) were cultured in LB and supplements with aeration to an OD_600_ of 0.85. Cultures were centrifuged at 4,000 rpm at room temperature and resuspended in buffered saline containing 0.01% gelatin (BSG) [45]. Vaccine strains were orally administered to groups of mice at doses 1x10^9^, 1x10^8^ or 1x10^7^ CFU in 20 μl BSG. 20 μl of BSG was orally administered to control groups (mock immunized). Challenge strains UK-1 and 14028S were grown and administered similarly on day 35 post immunization at 1x10^9^ or 1x10^8^ CFU in 20 μl BSG. Animals were monitored closely twice a day for 30 days for signs of mortality and morbidity following lethal challenge. The actual vaccination and challenge doses administered were determined by dilution plating on LB agar with supplements.

### Assessment of antibody responses

Blood and vaginal washes were collected individually from immunized and control groups on the day of immunization, as well as week 1 and week 2 post immunization. 100 μl blood was collected by submandibular bleeding, and samples were incubated at 37°C for 1 h before separating the serum fractions by centrifugation at 13,000 rpm for 10 min in an Eppendorf 5415D digital tabletop microfuge (Marshall Scientific, NH). Vaginal secretions were collected as 50 μl BSG washes and stored at -20°C. Antibody responses were assayed by enzyme-linked immunosorbent assay (ELISA) [46]. *Salmonella* serovar Typhimurium outer membrane proteins (SOMPs) were purified from χ9761 as described previously [47]. Purified lyophilized lipopolysaccharide (LPS) isolated from *S*. *enterica* serovar Typhimurium (O-4,5,12) was obtained from Sigma. LPS and SOMPs were used at 1 μg/ml and 2 μg/ml, respectively for sensitization of 96-well flat-bottom microtiter plates (Nalge Nunc. Rochester, NY, USA). They were then incubated overnight at 4°C. Sera were diluted starting from 1:200 for detection of total IgG, and 1:100 for IgG1 and IgG2a titers, before adding to individual wells in triplicate. Vaginal secretions were pooled and diluted 1:10 before adding 100 μl of sample to wells in triplicate. Biotinylated goat anti-mouse IgA (vaginal washes), IgG, IgG1 or IgG2a were diluted 1: 10,000 for detection of anti-LPS and anti-SOMP specific antibody titers.

### Assessment of cell-mediated responses

Antigen-presenting cells (APCs) were generated from spleens removed aseptically from seven-week-old naïve mice. Single-cell suspensions were counted by trypan blue exclusion [48], and treated with mitomycin C (5 μg/10^6^ cells) for 30 min in a 75-cm^2^ cell culture flask (Fisher Scientific, PA), incubated at 37°C in 5% CO_2_, followed by 2 h of incubation to obtain adherent APCs. 2 × 10^5^ APCs were seeded in each well (Corning 96-well TC-treated flat bottom microplates, Sigma), and infected with UV-inactivated UK-1 or 14028S (10 MOI) for 2 h. These infected APCs were cocultured for 72 h with T-lymphocytes obtained from vaccinated mice.

To obtain primed T-lymphocytes, 5 mice per group were orally immunized with 1x10^9^ CFU dose of UK-1(Δ*aroA*), 14028S(Δ*aroA*) or BSG (control). The mice were euthanized on day 35. Spleens were disrupted to create single-cell suspensions. These suspensions were treated with RBC lysing buffer (Sigma, MO) at 37°C for 5 min to lyse the erythrocytes. Cells were washed, and viability was assessed by trypan blue exclusion. These single cell suspensions were enriched using an EasySep mouse T-cell enrichment kit (Stemcell Technologies, Vancouver, Canada). 1 x 10^6^ cells per well were cocultured with UK-1 or 14028S-infected APCs. Following the coculture with APCs for 72 h, cytokine levels in the culture supernatants were assayed to examine APC-mediated T-cell activation.

### Colonization, coinfection and bacterial shedding

Groups of mice (5 mice per time point) were immunized orally with 1x10^9^ CFU doses of strains UK-1(Δ*aroA*) and 14028S(Δ*aroA*) or one of the two wild-type strains: χ4138 (UK-1 with nalidixic acid resistance) [43] and 14028S (with pACYC184 plasmid–for chloramphenicol and tetracycline resistance [44]). Spleen, liver, Peyer’s patches, mesenteric lymph nodes (MLN), cecum, and ileum were removed on days 3 and 6 following immunization or challenge. Bacterial counts in the homogenized tissues were determined by dilution plating on MacConkey plates containing 1% lactose and appropriate antibiotics when required. 2 μg/ml each of PABA and DHB were also added to the MacConkey plates when determining UK-1(Δ*aroA*) and 14028S(Δ*aroA*) counts. Colonies obtained were screened for dependence on PABA and DHB by patching on LB agar ± PABA and DHB. For burden studies, mice were immunized with UK-1(Δ*aroA*) or 14028S(Δ*aroA*) and challenged with UK-1 on day 35 post immunization. Bacteria were enumerated in spleen, liver, and Peyer’s patches on day 6 post challenge.

Wild-type strains were also administered in pairs to evaluate the ability of UK-1 to colonize in the presence of the less-virulent 14028S. Bacteria were enumerated in spleen, liver, Peyer’s patches, (MLN), cecum, and ileum on days 3 and 6 after challenge.

Fecal pellets were collected from mice immunized with strains UK-1(Δ*aroA*) or 14028S(Δ*aroA*) on days 1, 2, 3, 7 and 10 after immunization for enumeration of bacterial shedding post vaccination. Colonies obtained on MacConkey plates with 1% lactose, PABA, and DHB (2 μg/ml) were screened for dependence on PABA and DHB by patching on LB agar ± PABA and DHB.

### Statistics

Statistical analyses were performed by using the GraphPad Prism 5 software package (Graph Software, San Diego, CA). Log-rank (Mantel-Cox) test was applied to compare survival curves following oral challenge. Antibody responses as well as bacterial titers in organs and fecal pellets were expressed as means ± standard deviations. One-way ANOVA test was used to evaluate differences in antibody titers between various groups of immunized mice. Mann-Whitney U test was used to compare bacterial titers in corresponding organs. Differences were considered significant at a *P* value of ≤0.05. *P ≤ 0.05, **P ≤ 0.01, ***P ≤ 0.001, ****P ≤ 0.0001. When required, data were transformed to log_10_ or log_2_ prior to these calculations.

## Supporting information

S1 Fig Schematic of immunization regimen. BALB/c mice (*n* = 10 per immunization dose) were orally immunized with UK-1(Δ*aroA*) or 14028S(Δ*aroA*) at doses 1x10^9^, 1x10^8^ or 1x10^7^ CFU in 20 μl BSG. Mice were also mock-vaccinated with 20 μl BSG. Blood and vaginal washes were collected on day 14 and day 28 post immunization for evaluating antibody responses against LPS and SOMP. Oral challenge at doses 1x10^9^ or 1x10^8^ CFU were performed with UK-1 or 14028S 35 days after immunization. Mice were monitored for mortality and signs of morbidity for 30 days after challenge.
(TIFF)

S2 Fig Anti-SOMP and anti-LPS IgG subtype titers in mice. Serum IgG1 and IgG2a responses to (A) LPS in UK-1(Δ*aroA*)-immunized mice, (B) LPS in 14028S(Δ*aroA*)-immunized mice, (C) SOMP in UK-1(Δ*aroA*)-immunized mice, and (D) SOMP in 14028S(Δ*aroA*)-immunized mice were determined by ELISA at 2 and 4 weeks after oral immunization in sera and vaginal washes from BALB/c mice (*n* = 6).
(TIFF)

S3 Fig IL-4 and IL-12 secretion following APC-mediated T-cell activation. T-lymphocytes obtained from BALB/c mice (*n* = 5) orally immunized with UK-1(Δ*aroA*), 14028S(Δ*aroA*) or BSG (mock) were co-incubated with respective UV-inactivated UK-1 or 14028S-infected and mitomycin C—treated APCs. APCs treated with an unrelated antigen, HEL, were also included for comparison. The co-cultures were incubated for 72 h and supernatants were collected for determination of (A) IL-4, and (B) IL-12.
(TIFF)

S4 Fig Colonization with UK-1 and 14028S. BALB/c mice were orally challenged with 1 x 10^9^ CFU of UK-1(Nal^r^) or 14028S(Cm^r^ Tc^r^). Groups of mice (n = 5 per group) were euthanized on days 3 and 6 post challenge. (A) Spleen, (B) liver, (C) Peyer’s patches, (D) MLN, (E) ileum, and (F) cecum were collected to determine the bacterial burdens. No statistically significant difference was observed between challenge groups.
(TIFF)

S5 Fig Colonization with UK-1(Δ*aroA*) and 14028S(Δ*aroA*). BALB/c mice were orally immunized with 1 x 10^9^ CFU UK-1(Δ*aroA*) or 14028S(Δ*aroA*). Groups of mice (n = 5 per group) were euthanized on days 3 and 6 post challenge. (A) Spleen, (B) liver, (C) Peyer’s patches, (D) MLN, (E) ileum, and (F) cecum were collected for enumeration of bacteria.
(TIFF)
