# Supplementary figures and images for: Comparative evaluation of Salmonella Typhimurium vaccines derived from UK-1 and 14028S: Importance of inherent virulence
Source: PLoS One. 2018 Sep 7;13(9):e0203526. doi: 10.1371/journal.pone.0203526 (PMC6130210; doi:10.1371/journal.pone.0203526)

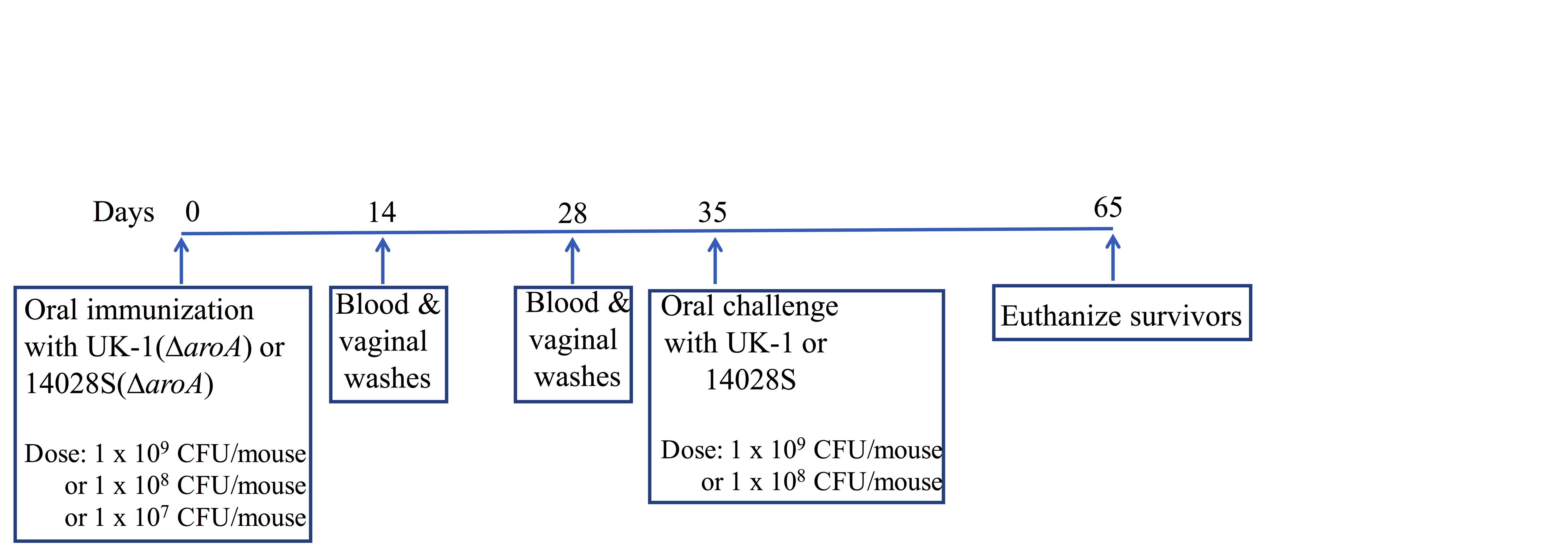

Supplement: S1 Fig — BALB/c mice (n = 10 per immunization dose) were orally immunized with UK-1(ΔaroA) or 14028S(ΔaroA) at doses 1x10^9^, 1x10^8^ or 1x10^7^ CFU in 20 μl BSG. Mice were also mock-vaccinated with 20 μl BSG. Blood and vaginal washes were collected on day 14 and day 28 post immunization for evaluating antibody responses against LPS and SOMP. Oral challenge at doses 1x10^9^ or 1x10^8^ CFU were performed with UK-1 or 14028S 35 days after immunization. Mice were monitored for mortality and signs of morbidity for 30 days after challenge. (TIFF) [file pone.0203526.s001.tiff]

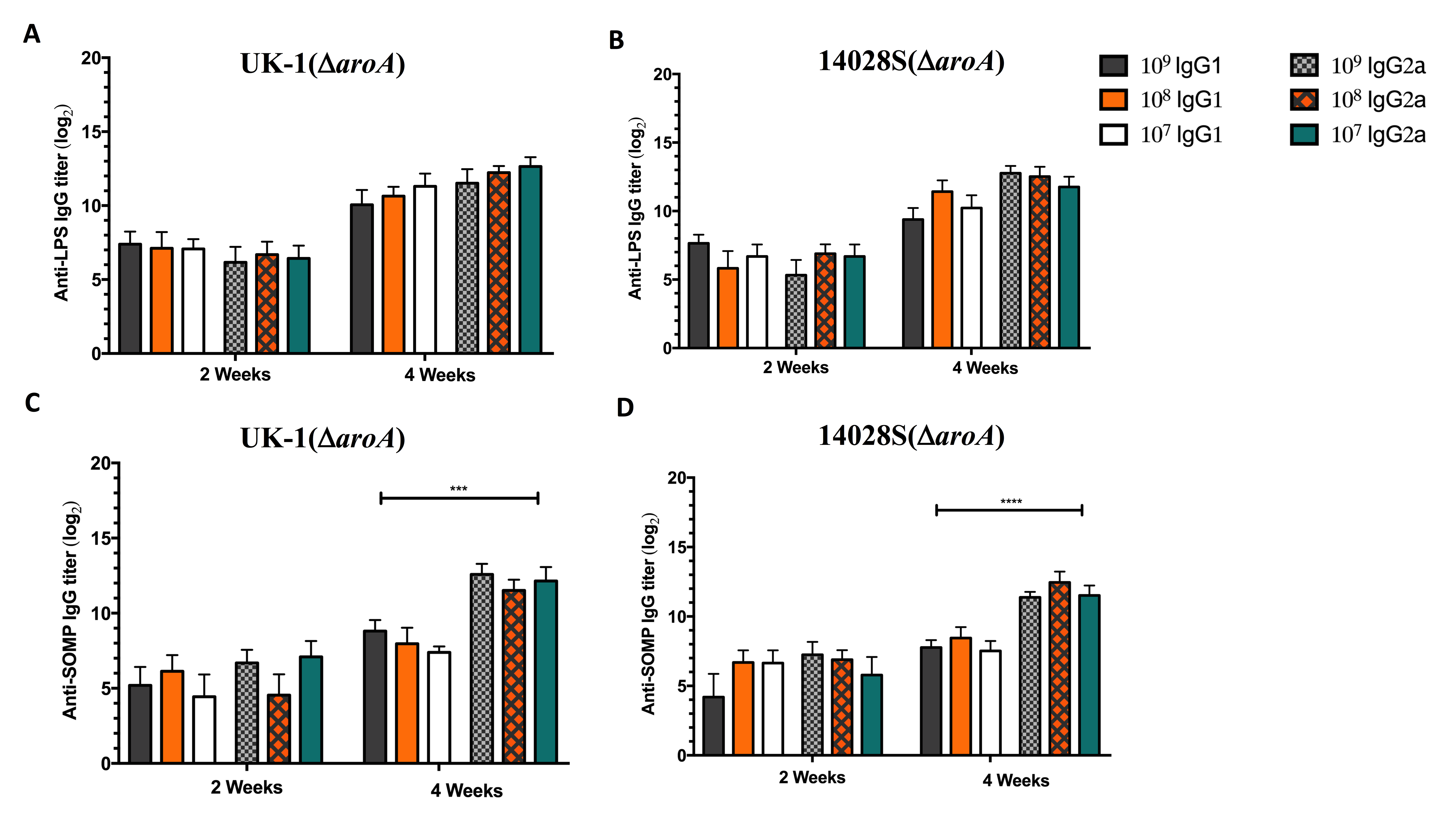

Supplement: S2 Fig — Serum IgG1 and IgG2a responses to (A) LPS in UK-1(ΔaroA)-immunized mice, (B) LPS in 14028S(ΔaroA)-immunized mice, (C) SOMP in UK-1(ΔaroA)-immunized mice, and (D) SOMP in 14028S(ΔaroA)-immunized mice were determined by ELISA at 2 and 4 weeks after oral immunization in sera and vaginal washes from BALB/c mice (n = 6). (TIFF) [file pone.0203526.s002.tiff]

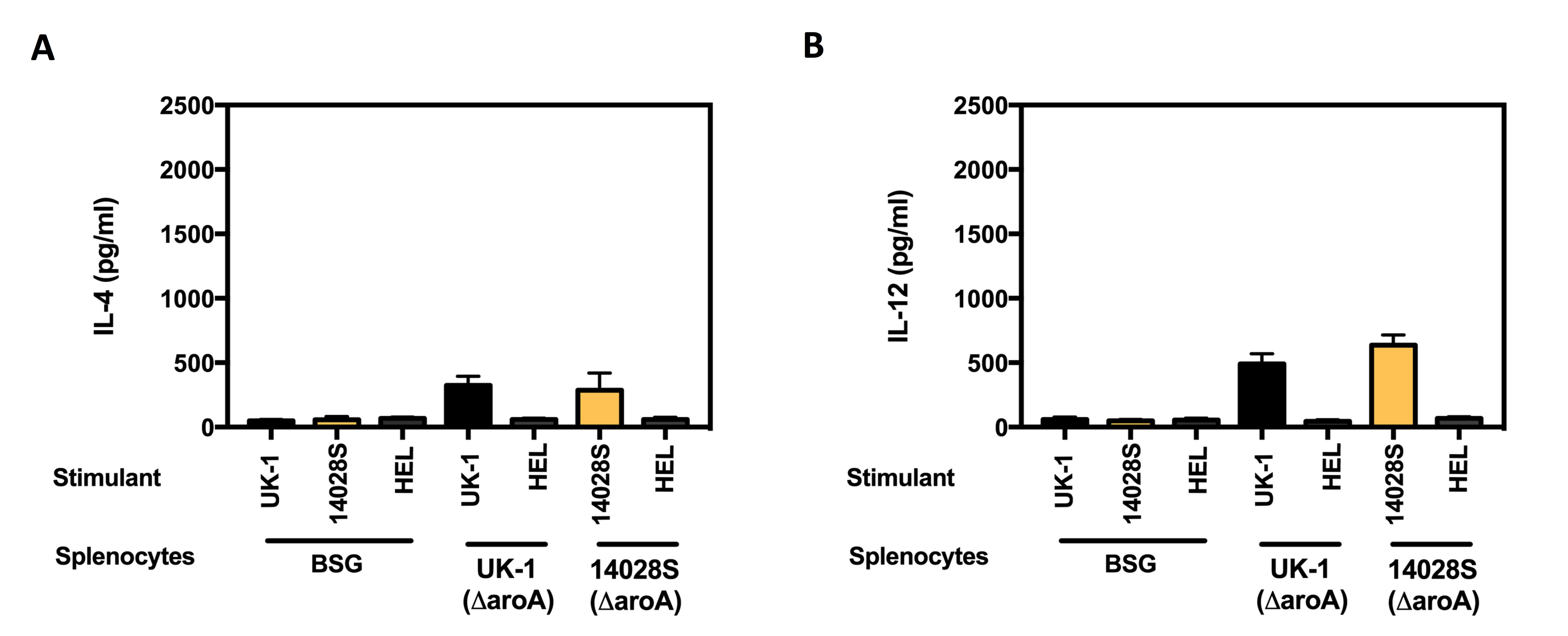

Supplement: S3 Fig — T-lymphocytes obtained from BALB/c mice (n = 5) orally immunized with UK-1(ΔaroA), 14028S(ΔaroA) or BSG (mock) were co-incubated with respective UV-inactivated UK-1 or 14028S-infected and mitomycin C—treated APCs. APCs treated with an unrelated antigen, HEL, were also included for comparison. The co-cultures were incubated for 72 h and supernatants were collected for determination of (A) IL-4, and (B) IL-12. (TIFF) [file pone.0203526.s003.tiff]

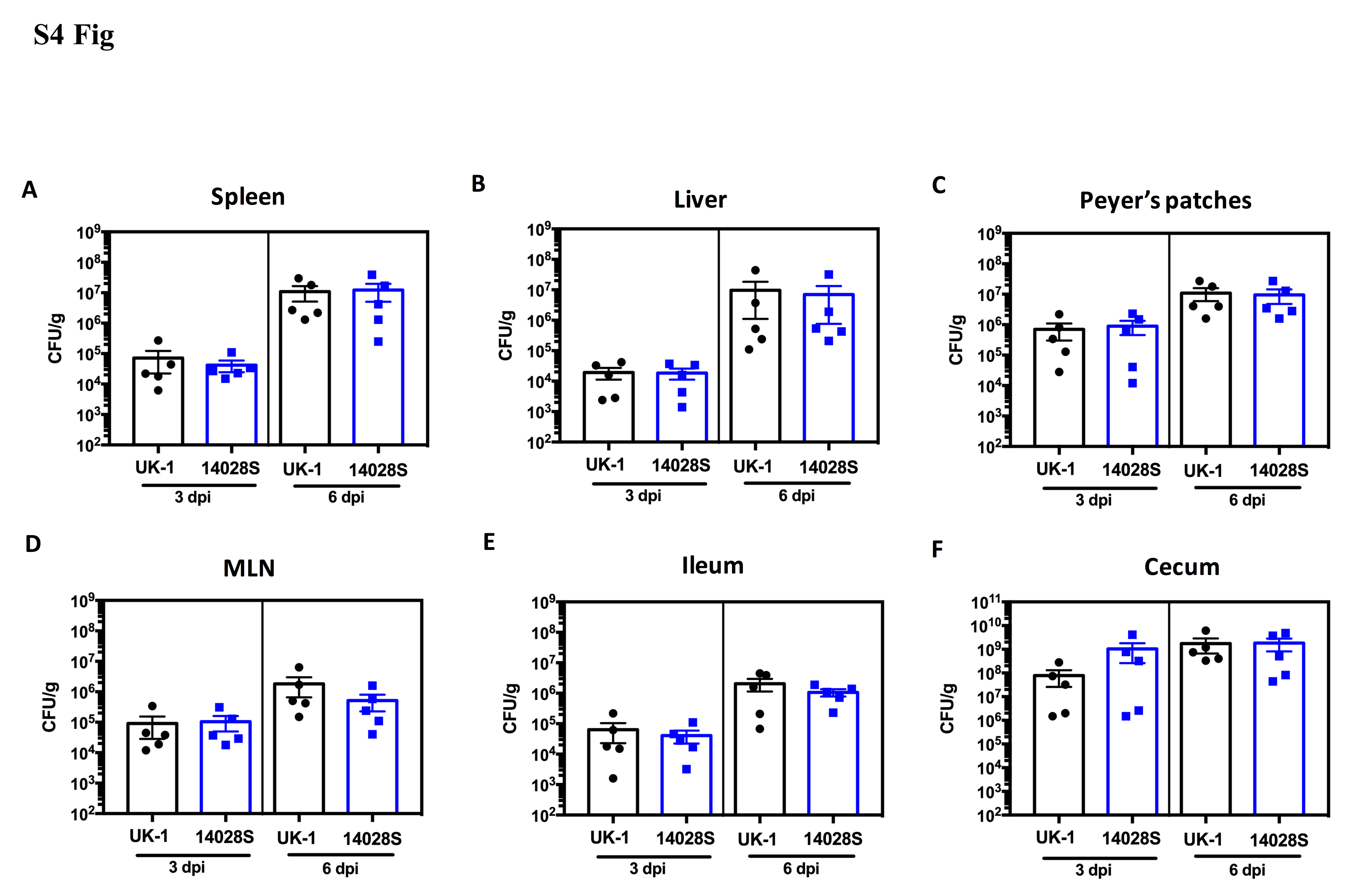

Supplement: S4 Fig — BALB/c mice were orally challenged with 1 x 10^9^ CFU of UK-1(Nalr) or 14028S(Cmr Tcr). Groups of mice (n = 5 per group) were euthanized on days 3 and 6 post challenge. (A) Spleen, (B) liver, (C) Peyer’s patches, (D) MLN, (E) ileum, and (F) cecum were collected to determine the bacterial burdens. No statistically significant difference was observed between challenge groups. (TIFF) [file pone.0203526.s004.tiff]

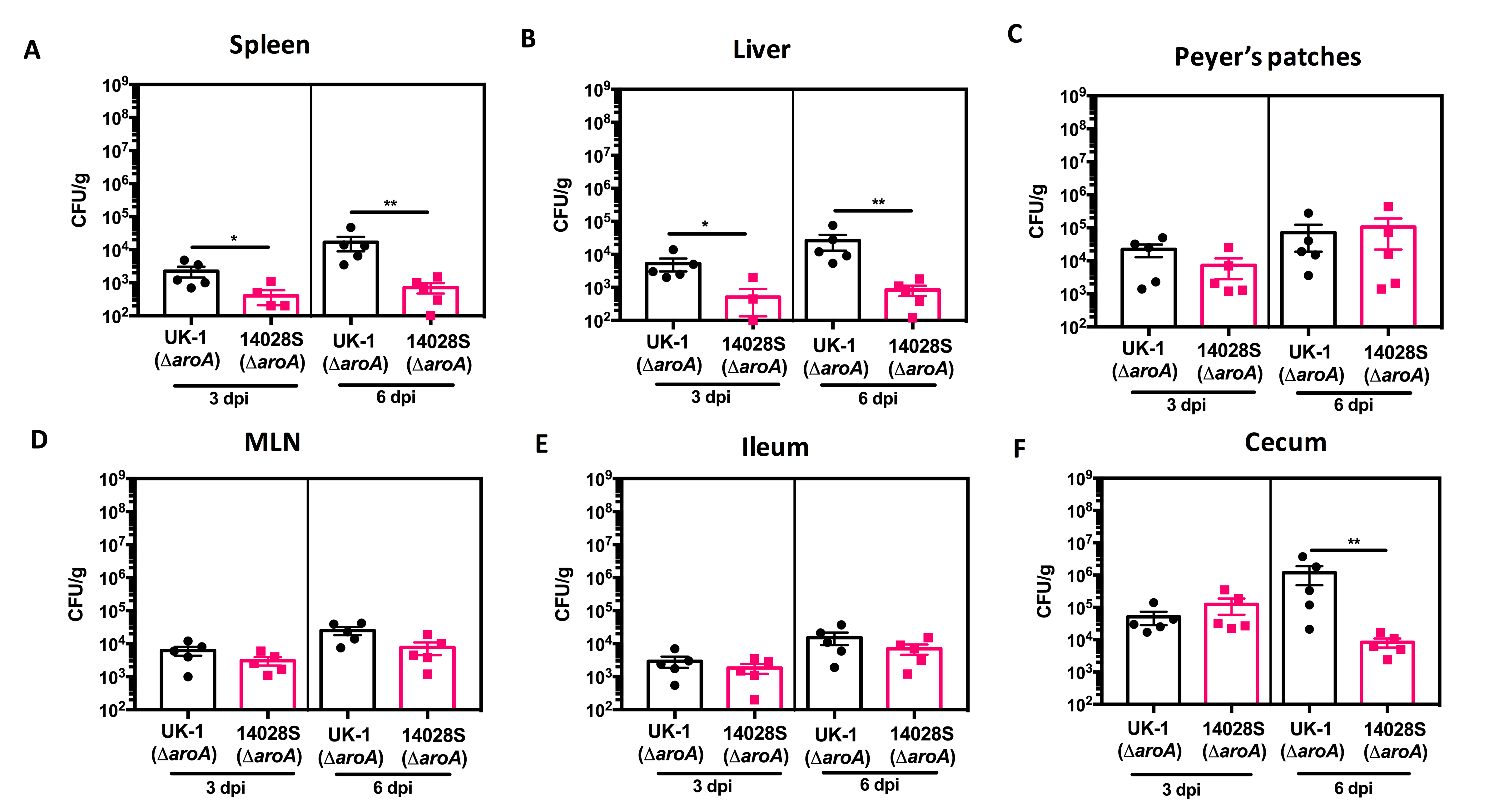

Supplement: S5 Fig — BALB/c mice were orally immunized with 1 x 10^9^ CFU UK-1(ΔaroA) or 14028S(ΔaroA). Groups of mice (n = 5 per group) were euthanized on days 3 and 6 post challenge. (A) Spleen, (B) liver, (C) Peyer’s patches, (D) MLN, (E) ileum, and (F) cecum were collected for enumeration of bacteria. (TIFF) [file pone.0203526.s005.tiff]
